# Child Spacing and Fertility Planning Behavior Among Women in Mana District, Jimma Zone, South West Ethiopia

**DOI:** 10.4314/ejhs.v20i2.69433

**Published:** 2010-07

**Authors:** Yohannes Dibaba

**Affiliations:** Department of Population and Family Health, Jimma University

**Keywords:** unintended pregnancy, child spacing, Mana, high risk

## Abstract

**Background:**

Short birth intervals and unintended pregnancies pose serious health risks to mothers and their infants by causing unnecessary high risk of pregnancy related complications and self induced abortions. The objective of the study was to assess the child spacing and fertility planning behavior of women in Mana district, Jimma zone.

**Methods:**

A cross-sectional survey was conducted from July 18 – August 17, 2008 on 645 women who had a live birth in the three years prior to the survey. A simple random sampling technique was used to identify eligible women. A pre-tested structured questionnaire was used for data collection. Data were analyzed using SPSS for windows version 15. Frequency distributions, cross-tabulation, and logistic regression analysis were performed.

**Results:**

Analysis of birth intervals for women with non first births showed that 27% of births occurred within less than 24 months after a previous birth, showing that a considerable proportion of births were not adequately spaced to promote maternal and child health. About 39% of women reported that their recent pregnancy was unintended. Women with unintended pregnancy are more likely to be illiterate (OR=1.85,95%CI,1.23–2.79), have four or more living children(OR=2.77,95% CI,1.77–4.33), had a previous birth interval of less than 24 months(OR=1.78,95% CI(1.19–2.69), have never used contraception (OR=4.53, 95% CI, 3.05–6.75) and did not desire any more children (OR=1.84, 95% CI, 1.23–2.76).

**Conclusion:**

The study showed that an inadequate child spacing and high level of unintended pregnancy among considerable proportion of the study population. Unintended pregnancy and short birth intervals can pose serious health risks to mothers and their infants by causing unnecessary high risk of pregnancy related complications. Thus, improving access to safe and voluntary family planning counseling and services is essential to reduce the high level of unintended pregnancy and short birth intervals.

## Introduction

Maternal and child mortality remains a major health problem. Of the estimated 535,900 maternal deaths and 9.2 million child deaths that occur annually in the world, about 99% occurs in the developing world (1, 2). The Ethiopian Demographic and Health Survey (DHS, 2005) showed 673 maternal deaths occur for every 100,000 live births and 123 under five deaths per 1000 births annually (3). Although timing and spacing of pregnancy is important for the health and survival of the newborn and the mother, unintended pregnancy and short birth interval are still among the contributing factors for the high maternal and child mortality in developing countries like Ethiopia.

The benefits of pregnancy planning and child spacing on maternal, infant and child health has been well documented (4–6). Researches have shown that family planning can reduce about 25% to 40% of maternal deaths by preventing unplanned and unwanted pregnancies, and about 10% of child deaths by eliminating inter-birth intervals of less than two years (7, 8).For many years, studies demonstrated that when mothers space births at least 2 years apart, their children are more likely to survive and to be healthy. Infants spaced at least 2 years apart are also less likely to be premature, of low birth weight, and to be malnourished (4, 6, 8). Recent studies also showed longer intervals are even better for infant and maternal survival and health than the two year interval earlier suggested. A study on the relationship between pregnancy intervals and Perinatal Mortality showed that children born 3 to 5 years after a previous birth are about 2.5 times more likely to survive than children born before 2 years (4). Similarly, studies on maternal mortality and morbidity associated with pregnancy interval showed birth intervals of 3 to 5 years are healthier for mothers too (6,10). Accordingly, the World Health Organization (WHO) technical consultation on birth spacing recommended a birth to conception interval of at least two years to reduce the risk of adverse maternal, perinatal and infant outcomes (9).

One of the outcomes of not using of family planning and unmet need is unintended pregnancy; pregnancy that is unwanted either at the time or at any time in the future and those that come sooner than desired (17).Worldwide, an estimated 210 million pregnancies occur annually, nearly 80 million are unintended and end in abortion, still birth and live birth. In developing countries, it was estimated that about 76 million unintended pregnancies occur annually, of which 34 million result in unplanned births and the remaining are interrupted by abortion and miscarriage (11–12). In Ethiopia, one third (31.5%) of all pregnancies were reported as either mistimed or unwanted (3).

Unintended pregnancy can pose serious health risks to mothers and their infants causing unnecessary complications. Thus, a better understanding of the magnitude and risk factors of unintended pregnancies will enable policy makers to reduce the obstacles that prevent families from having their desired number of children, and improve maternal and child health. But, few studies have examined the child spacing and fertility planning behavior of women in a rural setting in Ethiopia where fertility is too high and unintended pregnancies are widespread. Thus, the objective of this study was to examine the child spacing and fertility planning behavior of women in Mana District, Jimma zone.

## Methods and Materials

A cross-sectional study was conducted from July 18 to August 17, 2008 among women in Mana District to assess child spacing and fertility planning behavior of women. Mana District is located at about 370 kilometers to the south west of Addis Ababa and 22 kilometers to the West of Jimma Town, in Oromia Regional State. According to the 2007 Population and Housing Census of Ethiopia, the district had a total population of 149,661 in 2007 (13). Females constituted 49 % (73,443) of the district's population, of which females of child bearing age account for about 24% of the total female population. The district has 25 “kebeles” - the smallest administrative unit, of which 24 are rural kebeles. Nearly, 97% of the population of the district lives in rural areas.

The sample size of 645 was calculated using a formula for estimating a single population proportion assuming an expected prevalence of unintended pregnancy of 31% (3), 95% confidence level, a precision of .05 and a design effect of 2.

The Study Population was women who gave birth in the last three years prior to the survey and who resided in the district for more than 6 months. To identify the study units, six of the 24 rural *kebeles* were selected using lottery method. Household census and numbering was carried out to obtain a sampling frame (list of eligible women) before the actual data collection. A probability sample proportional to the population size was used to allocate the sample size to be interviewed from each *kebele*. The respondents were selected in each *Kebele* using simple random sampling techniques. At the household level, one eligible woman was selected for an interview. In the cases where there were more than one eligible respondent in the same household, one respondent was chosen using the lottery method.

A pre-tested interviewer administered questionnaire was used to collect the data. The questionnaire was first prepared in English and then translated to Oromo Language. Twelve female health extension workers were recruited, trained and employed as data collectors. They were trained on the content of the questionnaire, interviewing techniques, purpose of the study, and importance of privacy and confidentiality. Before conducting the main study, pre-test was carried out on 5% of the sample size selected from the near by *Kebeles* which were not included in the study.

The main outcome Variable of the study was women's fertility planning behavior; whether their previous pregnancy was intended or not intended. Unintended pregnancies include unwanted and mistimed pregnancies, pregnancies that are unwanted either at the time or at any time in the future and those that come sooner than desired as described in another study (18). In this study child spacing refers to the period between two consecutive live births, birth to birth interval. A birth interval of less than two years is considered as short birth interval.

Explanatory variables such as socio- economic, demographic factors, and reproductive health behavior of women were included to determine their association with the outcome variable.

Data were entered and analyzed using SPSS for windows version 15.0. Frequency distributions, cross-tabulation and logistic regression were used. Odds ratios with 95% confidence intervals were calculated using logistic regression models. Ethical clearance was obtained from the Research and Publication Office of Jimma University and official letter was written to the district and *kebele* administrators for permission to conduct the study. An informed verbal consent was obtained from each respondent.

## Results

A total of 627 women of aged 15 – 49 years were interviewed, with a response rate of 96.6%. More than half of the respondents, 352 (56.1%) were in the age range of 25–34 years, and the mean age of the respondents was 28 years (SD ±5.48). The majority of the respondents were Oromo, 577(92.0%), Muslim, 591(94.3%) and married, 608(97.0%). About two-third 445 (71.0%) of the respondents were illiterate while the remaining one third have attained a primary and secondary level of education. The majority of the respondents were housewives, 422 (67.4%) followed by farmers, 179 (28.5%) and traders, 18(2.9%), respectively. Four hundred nine (65%) of households own radio, while 179 (28.5%) have access to piped water supply (table 1).

**Table 1:** Socio-demographic characteristics of study participants, Mana district, Jimma Zone, 2008 (n=627).

Characteristics		Frequency (n=627)	percent
Age			
	15–24	145	23.1
	25–34	352	56.1
	35–49	130	20.7
Age at first Marriage			
	Below 18 years	321	51.2
	18 and above	306	48.8
Ethnicity			
	Oromo	577	92.0
	Amhara	11	1.8
	Dawro	14	2.2
	Kefa	5	0.8
	Others	20	3.2
Religion			
	Muslim	591	94.3
	Orthodox	30	4.8
	Protestant	6	1.0
Marital status			
	Currently married	608	97.0
	Widowed	10	1.6
	Divorced	7	1.1
	separated	2	0.3
Education			
	No formal education	445	71.0
	Primary(1–8)	170	27.1
	Secondary and above	12	1.9
Occupation			
	Housewife	422	67.4
	Farmer	179	28.5
	Trader	18	2.9
	others	8	1.2
Ownership of some household assets			
	Radio	409	65.2
	Television	20	3.2
	Piped water supply	179	28.5
	Electricity	101	16.1
	Telephone/Mobile	17	2.7

**Figure 1:**
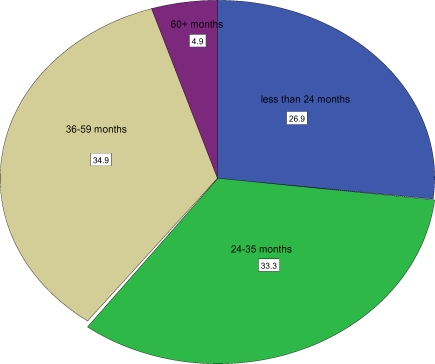
Birth interval in months in Mana District, 2008

The average number of children ever born was 4.02(±2.19). Analysis of birth intervals for women with non first births (at least two births) showed that 27% of births occurred within less than 24 months and 33% occurred between 24 and 35 months. Only 35% of births occurred between 36 and 59 months after a previous birth (fig 1). Regarding fertility planning, 382 (61%) women reported that their last birth was wanted, 146(23.3%) said it was mistimed while the remaining 99(15.8%) stated their last child was not wanted. This means that the magnitude of unintended (mistimed and unwanted) pregnancy among the study population was 39.1%. With regard to fertility preferences, about 338 (54%) said they want more children, 206 (33%) did not want any more child, while the remaining 83(13%) did not decide. Six hundred eleven (97.4%) of the respondents knew some form of family planning, while 218 (34.8%) of women reported current use of family planning (Table 2).

**Table 2:** Reproductive and family planning behavior of women, Mana District, 2008

Characteristics		Frequency (n=627)	percent
Desired number of children			
	1–3		144	23.0
	4–5	268	42.7
	6+	201	32.1
	Up to God/Allah	14	2.2
Number of living children			
	1–3	309	49.3
	4–5	213	34.0
	6+	105	16.7
Fertility preferences			
	Need more child(ren)	338	53.9
	Do not need more child	206	32.9
	Undecided/up to God	83	13.2
Sex of index child			
	Male	318	50.7
	female	309	49.3
Wantedenss of last birth			
	Wanted	382	60.9
	Mistimed	146	23.3
	Unwanted	99	15.8
Knowledge of contraception			
	Yes	611	97.4
	No	16	2.6
Ever use of family planning			
	Yes	303	48.3
	No	324	51.7
Current use of contraception			
	Using	218	34.8
	Not using	409	65.2

On multivariate logistic regression, education, number of living children, fertility preferences of women, previous birth interval, sex of index child, and use of family planning were important predictors of unintended pregnancies(p<0.05). Women with no formal education are 1.85 times more likely to report their last birth as unintended (OR=1.85, 95% CI, 1.23–2.79) as compared to women with some formal education (primary and above). Women with 4 or more living children were 2.8 times more likely to report their most recent pregnancy as unintended as compared to women with 1–3 living children (OR, 2.77; 95% CI,1. 77–4.33). Similarly, considering the fertility preferences, women who do not want any more children are nearly two times more likely to report their previous birth as unintended as compared to those who wanted more children (OR,1.84;95% CI1.23–2.76). With regards to birth interval, women whose previous birth interval was less than two years were 1.78 more likely (OR,1.78;95% CI,1.19–2. 96) to report their previous pregnancy as unintended as compared to women with a previous birth interval of three or more years. Women who have never used family planning are four and half times more likely (OR, 4.53; 95% CI, 3.05 – 6.75) to report unintended pregnancy as compared to women who have used family planning (table 3).

**Table 3:** Paramer estimates from bivariate and multivariable logistic regression model predicting the probability of unintended Pregnancy, Mana District, Jimma Zone, 2008

Variables		intended	unintended	OR(CI) unadjusted	OR(CI) adjusted
Age (Years)					
	15–24	115(79.3)	30(20.7)	1	1
	25–34	206(58.5)	146(41.5)	2.72(1.73–4.28)	1.14(0.66–1.90)
	35–49	61(46.9)	69(53.1)	4.34(2.56–7.36)	0.88(0.44–1.76)
Education status					
	No formal education	252(56.5)	194(43.5)	1.96(1.35–2.85)	1.85(1.23–2.79)*
	Primary & above	130(71.8)	51(28.2)	1	1
Number of live children					
	1–3	241(78.0)	68(22.0)	1	1
	4–5	107(50.2)	106(49.8)	3.51(2.40–5.14)	2.77(1.77–4.33)*
	6+	34(32.4)	71(67.6)	7.40(4.54–12.08)	5.94(3.20–11.03)*
Sex of index child					
	Male	207(65.1)	111(34.9)	1	1
	Female	175(56.6)	134(43.4)	1.43(1.04–1.97)	1.63(1.27–2.59)*
Fertility preferences					
	want more child	233(73.3)	85(26.7)	1	1
	Does not want more child	113(45.9)	133(54.1)	3.23(2.27–4.59)	1.84(1.23–2.76)*
	undecided/up to God	36(57.1)	27(42.9)	2.06(1.18–3.59)	1.39 (0.76–2.53)
Ever use of family planning					
	Yes	235(77.6)	68(22.4)	1	1
	No	147(45.4)	177(54.6)	4.16(2.94–5.89)	4.53(3.05–6.75)*
Using family planning					
	Yes	147(67.4)	71(32.6)	1	1
	No	235(57.5)	174(42.5)	1.07(0.76–1.50)	1.65(1.10–2.51)*
Birth to birth interval(Month)					
	< 24	97(51.9)	90(48.1)	1.94(1.32–2.84)	1.78(1.19–2.69)*
	24–35	90(59.2)	62(40.8)	1.44(0.96– 2.17)	1.38(0.89–2.15)
	≥36	195(67.7)	93(32.3)	1	1

* Significant at P <0.05

## Discussion

This study found out that there is a high level of unintended pregnancy among the study population. Thirty nine (39) percent of births were unintended, while the remaining 61% were intended. The magnitude of unintended pregnancy observed in this study is slightly higher than that reported by the 2005 Ethiopian DHS for the national level of 31 % (2). Analysis of DHS data from developing countries has showed that the magnitude of unintended pregnancy in developing countries ranges from 14 % to 62% of pregnancies (17). The relatively higher level of unintended pregnancy observed in this study, as compared to the report of EDHS, which may be related to differences in the study area, where the present study focused on rural women only. A larger proportion (59.6%) of the unintended pregnancy reported in this study is those which happened earlier than wanted (mistimed pregnancy) than those not wanted at all showing that most pregnancies occur sooner than women wanted.

Analysis of birth intervals for women with non first births showed that 27% of births occurred within less than 24 months, 33% occurred between 24 and 35 months and only 35% of births occurred between 36 and 59 months after a previous birth. The proportion of women with a birth interval of less than 24 months (27%) is relatively higher than the 2005 EDHS finding of 21%. This shows that a considerable proportion of births to women in this study are not adequately spaced to protect maternal and newborn health. The WHO technical consultation group on birth spacing advised an interval of at least 24 months before attempting the next pregnancy, in order to reduce the risk of adverse maternal, perinatal and infant outcomes (9). Recent researches have shown that spacing births 3 to 5 years apart increases children's and mothers chances of survival and health(4–7).

We found out that unintended pregnancy is significantly associated with women's education, number of living children, fertility preferences of women, birth interval, sex of index child and use of family planning. Women with no formal education are more likely to report their last birth as unintended as compared to women with some formal education. Such an association between educational status and fertility planning has been observed in previous studies in Ghana and Nigeria where it was found that educated women are less likely to report unintended pregnancy than uneducated women (14, 15). This could be due to the fact that educated women have better access to family planning information and services than uneducated women.

The number of living children a woman has is also associated with women's experience of unintended pregnancy. Women with large number of living children are more likely to report their previous birth as unintended. This may be due to the fact that women who have attained their desired number of children will regard any additional child as unwanted (19, 20). Similarly, considering the fertility preferences of women, women who do not want any more children are nearly two times more likely to report their previous birth as unintended as compared to those who want more children. Studies from Nigeria (15) and Ghana (14) have also reported that higher parity is associated with women's experiences of unintended pregnancy. The sex of index child and the length of birth interval are also important. Women whose index child was female were more likely to report their recent pregnancy as unintended as compared to women with a male child. With regards to birth interval, women whose previous birth interval was less than two years were more likely to report their previous pregnancy as unintended as compared to women with a previous birth interval of three or more years. Other studies have also shown that pregnancies which happen sooner after a previous birth are more likely to be unintended (12, 14, 19).

Use of Family planning is another important variable associated with unintended pregnancy. Women who have never used family planning were four and half times more likely to report unintended pregnancy as compared to women who have used family planning. It is known that non use of family planning and contraceptive failure are among the main causes of unintended pregnancy (17). It was reported that 78% of unwanted pregnancies were attributable to contraceptive non use, incorrect use, or method failure in Ethiopia (18). Many other similar studies have reported the effect of contraceptive non use on pregnancy planning behavior of women (14,15,19)

This study showed a high prevalence of unintended pregnancy among the study population, particularly among women with no education, women with large number of living children and among women who have never used family planning. The study also found out that a considerable proportion of births to women in this study are not adequately spaced to protect maternal and newborn health. Unintended pregnancy and short birth intervals pose serious health risks to mothers and their infants by causing high risk of pregnancy and related complications. Given the fact that current use of family planning is low, it is essential to improve access to family planning counseling and services to reduce unintended pregnancy.
